# 4-(5-Bromo-2-hydroxy­phen­yl)but-3-ene-2-one

**DOI:** 10.1107/S1600536809009386

**Published:** 2009-03-19

**Authors:** Afsaneh Zonouzi, Zakieh Izakiana, Hossein Rahmani, Seik Weng Ng

**Affiliations:** a Department of Chemistry, College of Science, University of Tehran, PO Box 13145-143, Tehran, Iran; b Institute of Chemical Industries, Iranian Research Organization for Science and Technology, PO Box 15815-358, Tehran, Iran; c Department of Chemistry, University of Malaya, 50603 Kuala Lumpur, Malaysia

## Abstract

The molecule of the title compound, C_10_H_9_BrO_2_, a doubly conjugated unsaturated ketone, is almost planar (r.m.s. deviation of the non-H atoms = 0.039 Å). In the crystal structure, two mol­ecules are linked across a centre of inversion to form a hydrogen-bonded dimer by way of two O—H⋯O links.

## Related literature

The reactivity of doubly conjugated unsaturated ketones has been known for a long time; see: Buck & Heilbron (1922 ▶); Marvel *et al.* (1953 ▶). Their utility is discussed by Trost & Fleming (1991 ▶).
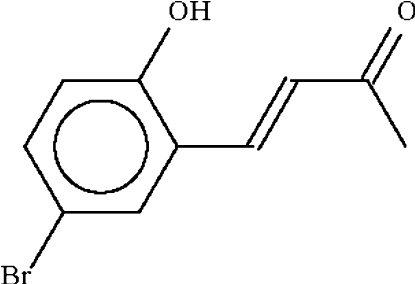


## Experimental

### 

#### Crystal data

C_10_H_9_BrO_2_
*M* *_r_* = 241.08
Triclinic, 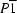

*a* = 5.8619 (2) Å
*b* = 7.7495 (2) Å
*c* = 10.9601 (3) Å
α = 106.432 (2)°
β = 104.548 (2)°
γ = 94.468 (2)°
*V* = 456.25 (2) Å^3^
*Z* = 2
Mo *K*α radiation
μ = 4.47 mm^−1^
*T* = 123 K
0.40 × 0.10 × 0.02 mm

#### Data collection

Bruker SMART APEX diffractometer
Absorption correction: multi-scan (*SADABS*; Sheldrick, 1996 ▶) *T*_min_ = 0.268, *T*_max_ = 0.916
3659 measured reflections
2040 independent reflections
1797 reflections with *I* > 2σ(*I*)
*R*_int_ = 0.025

#### Refinement

*R*[*F* ^2^ > 2σ(*F* ^2^)] = 0.031
*wR*(*F* ^2^) = 0.074
*S* = 1.04
2040 reflections
123 parameters
1 restraint
H atoms treated by a mixture of independent and constrained refinement
Δρ_max_ = 0.66 e Å^−3^
Δρ_min_ = −0.58 e Å^−3^

### 

Data collection: *APEX2* (Bruker, 2008 ▶); cell refinement: *SAINT* (Bruker, 2008 ▶); data reduction: *SAINT*; program(s) used to solve structure: *SHELXS97* (Sheldrick, 2008 ▶); program(s) used to refine structure: *SHELXL97* (Sheldrick, 2008 ▶); molecular graphics: *X-SEED* (Barbour, 2001 ▶); software used to prepare material for publication: *publCIF* (Westrip, 2009 ▶).

## Supplementary Material

Crystal structure: contains datablocks global, I. DOI: 10.1107/S1600536809009386/bt2898sup1.cif

Structure factors: contains datablocks I. DOI: 10.1107/S1600536809009386/bt2898Isup2.hkl

Additional supplementary materials:  crystallographic information; 3D view; checkCIF report

## Figures and Tables

**Table 1:** Hydrogen-bond geometry (Å, °)

*D*—H⋯*A*	*D*—H	H⋯*A*	*D*⋯*A*	*D*—H⋯*A*
O1—H1⋯O2^i^	0.83 (1)	1.87 (1)	2.689 (3)	168 (4)

Symmetry code: (i) 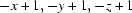.

**Fig. 1.**
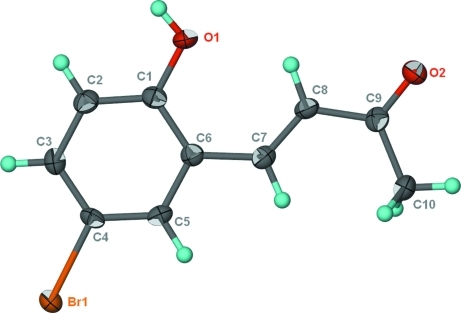
Anisotropic displacement ellisoid plot (Barbour, 2001) of C10H9BrNO2; probability levels are set at 70% and H-atoms are drawn as spheres of arbitrary radius.

## supplementary crystallographic information

### Experimental

To a stirred solution of 5-bromo-2-hydroxy-benzaldehyde (1.01 g, 5 mmol) in acetone (50 ml) a solution of sodium hydroxide (1.25 g, 6 mmol) in water (20 ml) was added. The reaction was stirred for another 6 h before being neutralized with strong hydrochloric acid to a pH of 6. The organic phase was washed by aqueous solution of sodium bisulfate (40%). After removing the solvent, the light green powder recrystallized from dichloromethane to give colorless plates (yield 85%, m.p. 421 K).

### Refinement

Carbon-bound H-atoms were placed in calculated positions (C–H 0.95 to 0.99 Å) and were included in the refinement in the riding model approximation, with *U*(H) set to 1.2 to 1.5*U*(C).

The hydroxy H-atom was located in a difference Fourier map, and was refined with a distance restraint of O–H 0.84±0.01 Å.

### Crystal data

**Table:**

C_10_H_9_BrO_2_	*Z* = 2
*M**_r_* = 241.08	*F*(000) = 240
Triclinic, *P*1	*D*_x_ = 1.755 Mg m^−^^3^
Hall symbol: -P 1	Mo *K*α radiation, λ = 0.71073 Å
*a* = 5.8619 (2) Å	Cell parameters from 1830 reflections
*b* = 7.7495 (2) Å	θ = 2.8–28.1°
*c* = 10.9601 (3) Å	µ = 4.47 mm^−^^1^
α = 106.432 (2)°	*T* = 123 K
β = 104.548 (2)°	Plate, colorless
γ = 94.468 (2)°	0.40 × 0.10 × 0.02 mm
*V* = 456.25 (2) Å^3^	

### Data collection

**Table:**

Bruker SMART APEX diffractometer	2040 independent reflections
Radiation source: fine-focus sealed tube	1797 reflections with *I* > 2σ(*I*)
graphite	*R*_int_ = 0.025
ω scans	θ_max_ = 27.5°, θ_min_ = 2.0°
Absorption correction: multi-scan (*SADABS*; Sheldrick, 1996)	*h* = −7→7
*T*_min_ = 0.268, *T*_max_ = 0.916	*k* = −10→9
3659 measured reflections	*l* = −14→14

### Refinement

**Table:**

Refinement on *F*^2^	Primary atom site location: structure-invariant direct methods
Least-squares matrix: full	Secondary atom site location: difference Fourier map
*R*[*F*^2^ > 2σ(*F*^2^)] = 0.031	Hydrogen site location: inferred from neighbouring sites
*wR*(*F*^2^) = 0.074	H atoms treated by a mixture of independent and constrained refinement
*S* = 1.04	*w* = 1/[σ^2^(*F*_o_^2^) + (0.0287*P*)^2^ + 0.4716*P*] where *P* = (*F*_o_^2^ + 2*F*_c_^2^)/3
2040 reflections	(Δ/σ)_max_ = 0.001
123 parameters	Δρ_max_ = 0.66 e Å^−^^3^
1 restraint	Δρ_min_ = −0.58 e Å^−^^3^

### Fractional atomic coordinates and isotropic or equivalent isotropic displacement parameters (Å^2^)

**Table:**

	*x*	*y*	*z*	*U*_iso_*/*U*_eq_	
Br1	0.69822 (5)	0.19383 (4)	−0.18504 (3)	0.02165 (11)	
O1	0.3724 (4)	0.3898 (3)	0.3090 (2)	0.0231 (5)	
H1	0.256 (4)	0.319 (4)	0.307 (4)	0.033 (11)*	
O2	0.9820 (4)	0.8201 (3)	0.6596 (2)	0.0226 (5)	
C1	0.4405 (5)	0.3456 (4)	0.1967 (3)	0.0173 (6)	
C2	0.2974 (5)	0.2178 (4)	0.0796 (3)	0.0188 (6)	
H2	0.1468	0.1611	0.0782	0.023*	
C3	0.3699 (5)	0.1715 (4)	−0.0348 (3)	0.0192 (6)	
H3	0.2722	0.0828	−0.1138	0.023*	
C4	0.5894 (5)	0.2583 (4)	−0.0309 (3)	0.0173 (6)	
C5	0.7326 (5)	0.3867 (4)	0.0822 (3)	0.0168 (6)	
H5	0.8812	0.4441	0.0815	0.020*	
C6	0.6617 (5)	0.4341 (4)	0.1992 (3)	0.0166 (6)	
C7	0.8291 (5)	0.5694 (4)	0.3152 (3)	0.0178 (6)	
H7	0.9703	0.6173	0.3004	0.021*	
C8	0.8136 (5)	0.6359 (4)	0.4390 (3)	0.0180 (6)	
H8	0.6731	0.5958	0.4582	0.022*	
C10	1.2237 (6)	0.8416 (4)	0.5192 (3)	0.0248 (7)	
H10A	1.3292	0.9287	0.6014	0.037*	
H10B	1.1786	0.9032	0.4521	0.037*	
H10C	1.3071	0.7412	0.4868	0.037*	
C9	1.0023 (5)	0.7671 (4)	0.5460 (3)	0.0177 (6)	

### Atomic displacement parameters (Å^2^)

**Table:**

	*U*^11^	*U*^22^	*U*^33^	*U*^12^	*U*^13^	*U*^23^
Br1	0.02299 (17)	0.02469 (17)	0.01502 (15)	−0.00029 (11)	0.00787 (11)	0.00169 (11)
O1	0.0206 (11)	0.0277 (12)	0.0199 (11)	−0.0035 (9)	0.0101 (9)	0.0037 (9)
O2	0.0263 (12)	0.0215 (11)	0.0178 (10)	−0.0001 (9)	0.0082 (9)	0.0025 (9)
C1	0.0201 (15)	0.0182 (14)	0.0161 (14)	0.0048 (11)	0.0067 (12)	0.0073 (11)
C2	0.0198 (15)	0.0189 (14)	0.0195 (14)	0.0013 (12)	0.0071 (12)	0.0080 (12)
C3	0.0196 (15)	0.0188 (14)	0.0160 (14)	0.0021 (12)	0.0023 (12)	0.0035 (11)
C4	0.0213 (15)	0.0173 (14)	0.0165 (13)	0.0068 (12)	0.0099 (12)	0.0054 (11)
C5	0.0147 (14)	0.0193 (14)	0.0189 (14)	0.0032 (11)	0.0066 (11)	0.0079 (11)
C6	0.0179 (14)	0.0157 (14)	0.0158 (13)	0.0031 (11)	0.0047 (11)	0.0047 (11)
C7	0.0157 (14)	0.0193 (14)	0.0189 (14)	0.0026 (11)	0.0043 (12)	0.0074 (12)
C8	0.0153 (14)	0.0181 (14)	0.0205 (14)	0.0011 (11)	0.0057 (12)	0.0056 (12)
C10	0.0215 (16)	0.0251 (16)	0.0223 (15)	−0.0054 (13)	0.0070 (13)	0.0005 (13)
C9	0.0205 (15)	0.0150 (14)	0.0179 (14)	0.0025 (11)	0.0059 (12)	0.0056 (11)

### Geometric parameters (Å, °)

**Table:**

Br1—C4	1.903 (3)	C5—C6	1.406 (4)
O1—C1	1.350 (3)	C5—H5	0.9500
O1—H1	0.834 (10)	C6—C7	1.461 (4)
O2—C9	1.235 (3)	C7—C8	1.338 (4)
C1—C2	1.394 (4)	C7—H7	0.9500
C1—C6	1.409 (4)	C8—C9	1.460 (4)
C2—C3	1.385 (4)	C8—H8	0.9500
C2—H2	0.9500	C10—C9	1.508 (4)
C3—C4	1.390 (4)	C10—H10A	0.9800
C3—H3	0.9500	C10—H10B	0.9800
C4—C5	1.371 (4)	C10—H10C	0.9800
			
C1—O1—H1	114 (3)	C5—C6—C7	116.5 (3)
O1—C1—C2	121.9 (3)	C1—C6—C7	125.4 (3)
O1—C1—C6	118.3 (3)	C8—C7—C6	129.8 (3)
C2—C1—C6	119.8 (3)	C8—C7—H7	115.1
C3—C2—C1	121.5 (3)	C6—C7—H7	115.1
C3—C2—H2	119.2	C7—C8—C9	123.1 (3)
C1—C2—H2	119.2	C7—C8—H8	118.5
C2—C3—C4	118.2 (3)	C9—C8—H8	118.5
C2—C3—H3	120.9	C9—C10—H10A	109.5
C4—C3—H3	120.9	C9—C10—H10B	109.5
C5—C4—C3	121.6 (3)	H10A—C10—H10B	109.5
C5—C4—Br1	119.0 (2)	C9—C10—H10C	109.5
C3—C4—Br1	119.3 (2)	H10A—C10—H10C	109.5
C4—C5—C6	120.7 (3)	H10B—C10—H10C	109.5
C4—C5—H5	119.6	O2—C9—C8	120.5 (3)
C6—C5—H5	119.6	O2—C9—C10	119.0 (3)
C5—C6—C1	118.1 (3)	C8—C9—C10	120.5 (3)
			
O1—C1—C2—C3	−179.2 (2)	O1—C1—C6—C5	179.6 (2)
C6—C1—C2—C3	1.6 (4)	C2—C1—C6—C5	−1.2 (4)
C1—C2—C3—C4	−1.0 (4)	O1—C1—C6—C7	1.2 (4)
C2—C3—C4—C5	0.0 (4)	C2—C1—C6—C7	−179.5 (3)
C2—C3—C4—Br1	178.5 (2)	C5—C6—C7—C8	−177.8 (3)
C3—C4—C5—C6	0.4 (4)	C1—C6—C7—C8	0.6 (5)
Br1—C4—C5—C6	−178.10 (19)	C6—C7—C8—C9	177.6 (3)
C4—C5—C6—C1	0.2 (4)	C7—C8—C9—O2	−177.8 (3)
C4—C5—C6—C7	178.7 (2)	C7—C8—C9—C10	2.9 (4)

### Hydrogen-bond geometry (Å, °)

**Table:**

*D*—H···*A*	*D*—H	H···*A*	*D*···*A*	*D*—H···*A*
O1—H1···O2^i^	0.83 (1)	1.87 (1)	2.689 (3)	168 (4)

Symmetry codes: (i) −*x*+1, −*y*+1, −*z*+1.
